# BJPsych Bulletin In Memoriam List August 2026

**DOI:** 10.1192/bjb.2026.10258

**Published:** 2026-08

Black, Alan Arnold, Brighton, UK, FRCPsych, 1981

Brown, Stephen, Birmingham, UK, FRCPsych Hon, 2000

Fleming, Christopher Francis, Edinburgh, UK, MRCPsych, 1974

Foley, Brian Mary, Sydney, Nova Scotia, Canada, MRCPsych, 1986

Franciosi, Paola Ginevra, London, UK, MRCPsych, 1983

Goodman, Robert Nicholas, London, UK, FRCPsych, 1997

Guly, Olivia Claire Ruth, Preston, UK, MRCPsych, 1986

H.R.H. The Duchess of Kent, Katharine Lucy Mary Worsley, London, UK, FRCPsych Hon, 1997

Howorth, Peter Willis, Ferryhill, UK, MRCPsych, 1996

Littlewood, Roland Martin, London, UK, FRCPsych, 1999

Masood, Mohammad, Birmingham, UK, MRCPsych, 1976

McGuffin, Peter, Cardiff, UK, FRCPsych, 1990

Munro, Alistair, Eastern Passage, Nova Scotia, Canada, FRCPsych, 1972

Ogundipe, Adenike Olasimbo, Preston, UK, Affiliate, 2016

Ramamurthy, Vathsala, Berkhamsted, UK, FRCPsych, 2003

Ross, Josiah, Lewes, UK, MRCPsych, 1972

Shirley, Stephanie, Henley-on-Thames, UK, FRCPsych Hon, 2017

Small, Margarey Smith Stuart, Edinburgh, UK, MRCPsych, 1972

Waddell, James Lindsay, Haddington, UK, MRCPsych, 1972

Whybrow, Peter Charles, Los Angeles, California, USA, FRCPsych, 1979

For practical reasons the journal publishes a limited number of obituaries, but other tributes and obituaries of College Members and Fellows are available on The Royal College of Psychiatrists’ website. We would encourage readers to visit https://www.rcpsych.ac.uk/members/obituaries.

